# Thanks to reviewers list 2024

**DOI:** 10.1080/0886022X.2024.2438445

**Published:** 2025-01-20

Abdalbary, Mohamed

Abdelaziz, Tarek

Abdel-Daim, Mohamed

Abdelrahman, Aly M.

Abd El-Samad, Lamia

Abdelzaher, Walaa Yehia

Abderrahim, Ezzeddine

Abdollahi, Ashkan

Abdul Salim, Sohail

Abosamak, Mohammed

Aboubakr, Mohamed

Abrahams, Alferso C.

Abramson, Matthew

Adedapo, Adeolu

Adel Abdel, Aziz Mohamed

Agarwal, Rajiv

Agostinho Cabrita, Ana De Lurdes

Ahmadi, Farokhlegha

Ahmed, Heba Mostafa

Ahmed, Mueen

Akgul, Emin

Aktas, Gulali

Aktas, Gulali

Akyol, Asiye

Alagöz, Selma

Alanio, Alexandre

Aldohayan, Abdullah

Alexander, Suceena

Alhwiesh, Abdullah

Ali, Ahlam

Ali, Hatem

Al-Mayouf, Sulaiman M

Al Musaimi, Othman

Alotaibi, Manal

Alp, Alper

Alrowaie, Fadel

Alshahrani, Saeed

Alsouqi, Aseel

Amador, Cristián A.

Amaechi, Prince Keshide

Amasyali, Akin Soner

Ambuehl, Patrice

Ameh, Idaomeh

Amirteimoury, Morteza

Amorim, Fabio Ferreira

Amornritvanich, Porntep

An, Won Suk

An, Xiuping

Andersen, Ulrik

Andreotti, Giuseppina

Anic, Branimir

Anraku, Makoto

Antar, Samar A

Antonic, Miha

Antonucci, Luca

Anumas, Suthiya

Ao, Guangyu

Aoun, Mabel

Apostolos, Anastasios

Arabzadeh Bahri, Razman

Arafat, Amr

Arslan, Mustafa

Artunc, Ferruh

Aryaeian, Naheed

Assadiasl, Sara

Assumpcao, Lia

Attakorah, Joseph

Aucella, Filippo

Audzeyenka, Irena

Auguste, Bourne L

Avramovski, Petar

Awobusuyi, Olugbenga

Ayar, Yavuz

Azer, Samy

Azevedo Mendes, Daniel

Aziz, Fahad

Azushima, Kengo

BábíčKová, Janka

Bai, Hualong

Bai, Mi

Bai, Xiaoyan

Balafa, Olga

Balbaa, Mahmoud

Banerjee, Santasree

Bantis, Christos

Bao, Hui

Barrera-Chimal, Jonatan

Barrera-Chimal, Jonatan

Bartosova, Maria

Batte, Anthony

Bayliss, George

Bayraktar, Necmi

Bayston, Roger

Bedi, Sonam

Bejrananda, Tanan

Bellocchio, Francesco

Bentall, Andrew

Berezin, Alexander

Berthelot, Laureline

Besarab, Anatole

Besiroglu, Huseyin

Bevc, Sebastjan

Bharati, Joyita

Bhat, Riyaz

Bhatt, Aditi

Bhimma, Rajendra

Bi, Shu-Hong

Bian, Luyan

Bianchi, Maria Eugenia Victoria

Bignon, Yohan

Bilgili, Beliz

Billany, Roseanne

Bin, Sofia

Bingöl ÖzakpıNar, Özlem

Blinder, Joshua J.

Bo, Yang

Bobot, Mickaël

Bolasco, Piergiorgio

Borghi, Claudio

Borulu, Ferhat

Boutin, Louis

Bowring, Mary G.

Boyd, J Gordon

Brandi, Lisbet

Brinar, Ivana Vukovic

Brind’Amour, Alexandre

Buckley, Leo F.

Bugajska, Jolanta

Bunout, Daniel

Burns, Kevin D.

Cabral, Bernardo Pereira

Cakalagaoglu, Fulya

Çakir, Murat

ÇakıR, Murat

C Albright Jr, Robert

Campo, Giuseppe Maurizio

Can, Özgür

Çanga, YiğIt

Canpolat, Ugur

Cantarelli, Chiara

Cantero-Recasens, Gerard

Cao, Xin-Xin

Cao, Yirui

Capusa, Cristina

Caroli, Anna

Casagrande, Giustina

Caturano, Alfredo

Caza, Tiffany N.

Cecere, Pasqualina

Çelik, Ersin

Chamunorwa, Joseph

Chang, Alexander R

Chang, Jer-Ming

Chang, Liang

Chao, Chia -Ter

Charitaki, Evangelia

Charitaki, Evangelia

Chau, Katrina

Chaudhary, Kunal

Chávez-Iñiguez, Jonathan S.

Chazot, Charles

Che, Ruochen

Chellappan, Anand

Chen, Chaosheng

Chen, Cheng

Chen, Chien-Liang

Chen, Chung-Ming

Chen, Fei-Fei

Chen, Fenglin

Chen, Haiyong

Chen, Hongbo

Chen, Jianping

Chen, Jiaxin

Chen, Jun-Feng

Chen, Junzhe

Chen, Liangmei

Chen, Liangwan

Chen, Lin

Chen, Lv-Yi

Chen, Tianxin

Chen, Xingxing

Chen, Yi-Ting

Chen, Yueqi

Chen, Yung-Hsiang

Chen, Yung-Ming

Chen, Zhaohong

Chen, Zhaowei

Chen, Zhimin

Cheng, Hong

Chewcharat, Api

Chiang, Heng-Pin

Chisavu, Lazar

Cianciolo, Giuseppe

Ciechanowski, Kazimierz

Cigarran, Secundino

Cobbaert, Christa M.

Colussi, Giacomo

Coronado, Jorge

Cueto Manzano, Alfonso Martín

Cui, Fangqiang

Cumhur Cure, Medine

Cüre, Erkan

Cybulsky, Andrey

Czirok, Szabina

Dabet, Moh’D Mohanad A. Al

Da Cruz, José Arnaldo Shiomi

Dampier, Carlton

Danesh, Farhad

Daoud, Ahmed

Darouich, Sihem

D’Ascenzo, Fabrizio

Dasgupta, Indranil Dasgupta

De Almeida, Edgar A. F.

Deepthi, Bobbity

Deger, Serpil Muge

Delanaye, Pierre

De Matos, Jorge Paulo Strogoff

Demir, Yeliz

De Pont, Anne-Cornelie

Desbiens, Louis-Charles

De Souza Freitas, Ana Tereza Vaz

Dessein, Patrick Hector

Dhakarwal, Pradeep

Di, Jia

Diena, Davide

Di Marco, Maurizio

Ding, Huihua

Ding, Nan

Ding, Weiwei

Ding, Ying

Dizaye, Kawa

Djaiani, George

Dogra, Pavitra Manu

Dong, Bingzi

Dong, Guiying

Dong, Lei

Dos Santos Rosa, Thiago

Drouven, Johannes W

Du, Xin

Duan, Jiayu

Duan, Jiayu

Duan, Lian

Duarte, Tayse

Duni, Anila

Dursun, Belda

Duzova, Halil

Dzekova-Vidimliski, Pavlina

DęBska-ŚLizień, Alicja

Ebrahim, Zarina

Echavarria, Raquel

Echavarria, Raquel

Eidi, Maryam

Ekart, Robert

Ekperikpe, Ubong S

Elatreisy, Adel

Elbarbary, Ahmed Hussieny

El-Khashab, Sahier

Ellervik, Christina

Elmorsy, Sarah

El Nakib, Gehad

Elshamaa, Manal F

Elshamy, Abdelsamed I.

Elsheemy, Mohammed

Engin, Mesut

Enikő, Király

Eren, Hilal

Eriguchi, Masahiro

Erken, Ertugrul

Erkut, Bilgehan

Eshraghi, Azadeh

Esposito, Pasquale

Famurewa, Ademola

Fan, Junming

Fan, Rui-Feng

Fan, Yanqin

Fang, Hui

Fang, Wei

Fang, Xiangdong

Farinha, Ana

Feng, Jianan

Feng, Song-Tao

Feng, Ye

Feng, Yue

Feriozzi, Sandro

Fila, Branko

Fitzgibbon, Wayne

Flack, John M.

Flammia, Rocco Simone

Flannery, Alexander H.

Formanowicz, Dorota

Forte, Maurizio

Franco Palacios, Carlos

Friedman, Allon N.

Frydman, Shir

Fu, Chen

Fu, Shuai

Fu, Wenxia

Fujikura, Tomoyuki

Fulop, Tibor

Fung, Winston

Gadalean, Florica

Gadola, Liliana

Galán-Llopis, Juan A

Gallazzini, Morgan

Gal-Oz, Amir

Gang, Sishir

Gao, Jiandong

Gao, Shuang

Gao, Xuxia

Gao, Yuan

Gao, Yue-Ming

Gao, Zhao

Garneata, Liliana

Gasperoni, Lorenzo

Gayrard, Nathalie

G Casanova, Alfredo

Ge, Ning

Ge, Yongchun

Gennari, F. John

George, Jacob

George, James F.

Germain, Michael

Germain, Michael

Ghai, Sandeep

Ghosh, Sanjib

Gil, Amnon

Gilani, Nima

Girón-Luque, Fernando

Gjorgjievski, Nikola

Gluhovschi, Cristina

Gluhovschi, Cristina

Godefroid, Nathalie

Goes, Miguel Angelo

Gollie, Jared M

Gomez, Gonzalo

Gomez, Hernando

Gondos, Tibor

Gong, Nianqiao

Gong, Xuezhong

Gonzalez-Quiroz, Marvin

Gopalakrishnan, Natarajan

Gou, Shen-Ju

GołęBiowski, Tomasz

Gracious, Noble

Gregorini, Marilena

Gr ković, Antun

Grubb, Anders

Gu, Min

Gu, Mingjia

Gu, Wan-Jie

Gu, Xiangchen

Guan, Shi-Yang

Gui, Yuan

Guldan, Mustafa

Gunay, Emrah

Güngör, Kültüral

Guo, Jia

Guo, Qiang

Guo, Weikang

Guo, Xiaojiao

Guo, Xiaoling

Guo, Zhi-Yong

Gupta, Pallav

Gupta, Sanjana

Haaskjold, Yngvar Lunde

Habas, Elmukhtar

Hadi, Amir

Hamasaki, Yoshifumi

Hamza, Ali

Han, Jiarui

Hangyu, Duan

Hao, Zhihui

Hao, Zongyao

Hariri, Geoffroy

Hariri, Geoffroy

Hartinger, Jan Miroslav

Haruhara, Kotaro

Hasbal, Nuri Baris

Hassan, Hicham I Cheikh

Hasson, Denise C

Hayek, Salim

He, Lian

He, Shubei

He, Weichun

He, Xuelin

He, Yunqiang

Headley, Samuel

Helvaci, Ozant

Herenda, Vedad

Hidaka, Sumi

Hirakawa, Makoto

Hiramitsu, Takahisa

Hiremath, Swapnil

Ho, Hsin-Jung

Hocher, Berthold

Hodson, Elisabeth M.

Hojs, Radovan

Holford, Nick

Honore, Patrick M

Horino, Taro

Horinouchi, Yuya

Hory, Bernard

Hota, Debasish

Hotter, Georgina

Hou, Guocun

Houssaini, Tarik Sqalli

Hsieh, Pei-Ling

Hsu, Shih-Ping

Hu, Fengqi

Hu, Huili

Hu, Zhangxue

Huang, Chien-Wei

Huang, Gang

Huang, Hongdong

Huang, Huangjingda

Huang, Jing

Huang, Kun

Huang, Xianghua

Huang, Yanping

Huba Endre, Zoltán

Hueso, Miguel

Hui, Wun Fung

Hull, Katherine L

Hung, Ming-Jui

Ichikawa, Daisuke

Ikenoue, Tatsuyoshi

Inal, Volkan

Ionescu, Dorin

Irish, Ashley

Isaac, Alfred Orina

Ishida, Akio

Ishikawa, Kazuhiro

Ishimori, Shingo

István, Dr. Bátai

Ito, Marie

Iwazu, Yoshitaka

J, Manjunath

Jacobs, Lucas

Jakopin, Eva

Jang, Hye Ryoun

Jankauskiene, Augustina

Jankowski, Jakub

Jaturapisanukul, Solos

Javaid, Muhammad Masoom

Jayasinghe, Ruwan

Jeloka, Tarun

J. Fazzari, Melissa

Jha, Alok

Jha, Pranaw Kumar

Jhang, Jia‐Fong

Jiaming, Su

Jiang, Xing-Ming

Jiang, Yamei

Jiang, Yuteng

Jin, Huimin

Jin, Jingjing

Jin, Shi

Jin, Xin

Jing, Danyang

Jing, Shuhui

Joki, Nobuhiko

Joseph, Adrien

Ju Guanqun

Kaan Akturk, Halis

Kablak-Ziembicka, Anna

Kacso, Ina Maria

Kaewput, Wisit

Kahraman, Dogan

Kakaei, Farzad

Kallidonis, Panagiotis

Kalra, Andrew

Kandir, Sinan

Kaneko, Shuzo

Kang, Seok Hui

Kanic, Vojko

Kannan, Lakshmi

Kara, Ali

Karimi, Mohammad-Hossein

Karimi, Zeinab

Kassianides, Xenophon

Katavetin, Pisut

Kawanami, Daiji

Kaypakli, Onur

Kaysi, Saleh

K. Bongoni, Anjan

Ke, Ben

Ke, Lu

Kefleyesus, Amaniel

Keles, Mustafa

Keller, Frieder X

Khajav, Mohammad Reza

Khan, Atlas

Khandelwal, Priyanka

Khandpur, Sukhanshi

Khokhar, Manoj

Khuller, Dinesh

Khurana, Sakshi

Killeen, Anthony A

Kim, Gheun-Ho

Kim, Hyunsuk

Kim, Yaerim

Kirsch, Alexander H.

Klinkhammer, Barbara Mara

Klisic, Aleksandra

Kliuk-Ben Bassat, Orit

Koc, Kubra

Kocak Yucel, Sibel

Koirala, Abbal

Koiwa, Fumihiko

Kokeny, Gabor

Kökény, Gábor

Kokol, Peter

Koleilat, Issam

Kong, Xianglei

Kong, Yonglun

Kopp, Jeffrey B.

Kopp, Jeffrey B.

Korucu, Berfu

Kosugi, Takaaki

Kouam, Arnaud Fondjo

Koyuncu, Sumeyra

Kreepala, Chatchai

Kuck, Kai

Kulkarni, Yogesh A.

Kuma, Akihiro

Kuma, Akihiro

Kumagai, Naonori

Kumar, Gurinder

Kumar, Jitendra

Kumar, Manoj

Kuo, Ko-Lin

Kuo, Yu-Ting

Kusirisin, Prit

Kusztal, Mariusz

Kwon, Osun

Kwong, Cheng Chak

KıSaoğLu, Hakan

Lafavers, Kaice

Lafavers, Kaice A

Lanktree, Matthew B.

Larsson, Anders

Lazarides, Miltos K.

Lazarus, Eilean Rathinasamy

Leavesley, David

Lee, Whanhee

Lei, Chong

Le Leu, Richard

Lengvárszky, Zsolt

Lenoir, Olivia

León-Román, Juan

Li, Aiqing

Li, Chunquan

Li, Dan

Li, Guanggang

Li, Junhua

Li, Lin-Lin

Li, Liuyang

Li, Longkai

Li, Min

Li, Qing

Li, Renxi

Li, Xiaodong

Li, Xudong

Li, Ya-Feng

Li, Yamei

Li, Yanmei

Li, Yi

Li, Yi

Li, Yong

Li, Zi

Liang, Mengjun

Liang, Yumei

Liebman, Scott E.

Lim, Cynthia Ciwei

Lim, Jeong Hoon

Lim, Lee-Moay

Lim, Lee-Moay

Lin, Cheng-Jui

Lin, Chia-Hsun

Lin, Jin

Lin, Liang-Yu

Lin, Shengwei

Lin, Yilu

Liu, Can

Liu, Chan

Liu, Chan-Min

Liu, Chao

Liu, Chengxuan

Liu, Feng

Liu, Hongfang

Liu, Hongxing

Liu, Huafeng

Liu, Limin

Liu, Lin

Liu, Mi

Liu, Ping

Liu, Qiang

Liu, Qi-Feng

Liu, Qinghua

Liu, Shuai

Liu, Suwen

Liu, Tongqiang

Liu, Wenhu

Liu, Wenrui

Liu, Wenwu

Liu, Xiaoqiang

Liu, Xing

Liu, Xingzheng

Liu, Yuxiang

Lockwood, Mark B.

Loizzi, Francesco

Lombardi, Gianmarco

Loupy, Alexandre

Lu, Baisong

Lu, Ping-Hsun

Lu, Renhua

Lu, Yuan-Qiang

Lubbad, Loay

Lucca, Leandro

Lugli, Gianmarco

Lundberg, Sigrid

Luo, Laimin

Luo, Xun

Luo, Yang

Lv, Chunyan

Ma, Dong Hong

Ma, Haoyang

Ma, Kuai

Ma, Kun-Ling

Ma, Xiao

Machado, Alisson

Machida, Shuichi

Maddatu, Judith

Magagnoli, Lorenza

Maithili Karpaga Selvi N, Nachimuthu

Malashicheva, Anna

Malhotra, Kiran Preet

Malik, Shafi

Mandolfo, Salvatore

Mansouri, Ladan

Mansur, Juliana

Mao, Weipu

Marghoob, Bahareh

Marquez-Sandoval, Fabiola

Martín-Del-Campo, Fabiola

Martinez-Castelao, Alberto

Martucci, Gennaro

MarčUn Varda, Nata a

Matsumoto, Yoshihiro

Mattana, Joseph

Maursetter, Laura

Maya, Maria

MałYszko, JołAnta

Mcadams, Meredith

Meier, Clemens M.

Meijers, Bjorn

Meléndez, Oliva Erika

Mendes, Marcela

Mendonca, Satish

Mendoza-Carrera, Francisco

Mengting, Li

Messchendorp, A. Lianne

Mestres, Gaspar

Metzger, Corinne E.

Metzinger, Laurent

Meyrier, Alain

Miao, Yun

Michishita, Ryoma

Michou, Vassiliki

Miedziaszczyk, MiłOsz

Milas, Oana

Milton, Talukder

Minami, Satoshi

Mirahmadi Eraghi, Mohammad

Mircescu, Gabriel

Mitchell, Brett M.

Mitchell, Constance A.

Miura, Kenichiro

Mizuno, Masashi

Mohebbati, Reza

Molnar, Miklos Z.

Moloney, Bróna

Momo, Kenji

Monardo, Paolo

Moniwa, Norihito

Montez-Rath, Maria E

Moreso, Francesc

Motwani, Rohini

Mualif, Siti Aisyah

Mubarak, Muhammed

Mubarak, Muhammed

Muciño Bermejo, María Jimena

Mueller, Thomas F

Mukhi, Dhanunjay

Mullane, Ryan

Munoz Mendoza, Jair

Muro, Manuel

Murt, Ahmet

Mushahar, Lily

Musial, Kinga

Mutluay, Ruya

Na, Ning

Nagahama, Kiyotaka

Nagai, Junya

Naito, Shokichi

Naito, Yoshiro

Nakano, Yusuke

Nakata, Takeshi

Nakayama, Shingo

Nakayama, Takashin

Nakhoul, Farid

Nans, Florens

Naqvi, Rubina

Nar, Rukiye

Nasr, Ashraf Youssef

Nateghi Haredasht, Fateme

Navar, L. Gabriel

Ndour, El Hadji Malick

Nerbass, Fabiana Baggio

Nesrallah, Gihad

Neumayr, Tara M

Ng, Jack Kc

Ng, Jack Kit Chung

Ng, Qin Xiang

Ni, Chunping

Ni, Shi-Hao

Ni, Wei-Jie

Nikuseva Marti, Tamara

Ning, Ding

Niyyar, Vandana Dua

Nlandu Mayamba, Yannick

Nochaiwong, Surapon

Nyariki, James Nyabuga

Oami, Takehiko

Odum, James

Ogawa, Tsuneo

Oguchi, Hideyo

Okamoto, Keisuke

Okamoto, Teppei

Olatunji, Opeyemi Joshua

Onder, Ali Mirza

Orieux, Arthur

Ossareh, Shahrzad

Ostojic, Jelena Nesovic

Ou, Tongwen

Ouyang, Chun

Oyagbemi, Ademola Adetokunbo

Ozcan, Seyda Gul

Özdemir, Aydan Akyüz

Öztürk, Savaş

Padanilam, Babu J.

Pakmanesh, Hamid

Panagiotou, Angeliki

Panma, Yuanita

Papanas, Nikolaos

Park, Hoon Suk

Park, Kyung-Min

Park, Meyeon

Park, Sangwoo

Parv, Florina

Parwani, Kirti

Pashang, Mina

Patel, Uday

Patial, Vikram

Pattharanitima, Pattharawin

Pei, Huaying

Peng, Hongquan

Peng, Xiang

Peppelenbosch, Arnoud G.

Perry, Amy

Perticone, Francesco

Pethő, Md, PhdÁkos

Petras, Dimitrios

Petrova, Iliyana

Piccin, Andrea

Piko, Nejc

Pinho, Natalia Alencar De

Piraino, Beth M

Pirozzi, Nicola

Politis, Emmanuelo

Posadas Salas, Maria Aurora

Prabhu, Attur Ravindra

Prasad, Narayan

Pressly, Jeffrey

PrkačIn, Ingrid

Pruijm, Menno

Pun, Patrick

Putzu, Alessandro

Qassam, Heider

Qi, Shiyong

Qi, Xingshun

Qiao, Xi

Qin, Wei

Qiu, Ming-Zhu

Radovic, Milan

Ragy, Omar

Rahman, Asadur

Rahman, Mahboob

Rai, Pradeep

Rai, Tatemitsu

Raja, Shahzad Gull

Rajasekaran, Arun

Rajput, Jaisingh

Rajsic, Sasa

Ramachandra Rao, Indu

Ramanathan, Kollengode

Ramanathan, Kumaresan

Ramírez, Victoria

Ramírez González, Victoria

Randjelovic, Pavle

Ranganathan, Dwarakanathan

Rao, Nitesh N

Rao, Pandurang S

Rasa, Kemal

Rathnayaka, R. M. M. K. Namal

Ravi, Katherine Scovner

Raynaud, Fabrice

Reeve, Jeff

Ren, Huiwen

Rezapour, Mohammad

Rhee, Harin

Ribitsch, Werner

Rivetti, Giulio

Roditi, Giles

Roehm, Bethany

Roetker, Nicholas S.

Rong, Ruiming

Ronsin, Charles

Roomizadeh, Peyman

Rosenstock, Jordan L.

Rossanti, Rini

Rostoker, Guy

Rotondi, Silverio

Roumelioti, Maria-Eleni

Ruengorn, Chidchanok

Rui-Feng, Wang

Rule, Andrew

Ruszkowski, Jakub

Sabath, Ernesto

Sadek, Kadry

Sagan, Jiri

Saha, Anshuman

Sahlawi, Muthana Al

Sahutoglu, Tuncay

Saito, Takao

Saka, Yosuke

Sakhi, Imene Bouchra

Salerno, Fabio R

Salve Coutinho Wolino, Karen

Samy, Mohamed

Sánchez-Navarro, Andrea

Sandal, Shaifali

Santos, Guilherme De Castro

Santos, Peter

Santovito, Donato

Santulli, Gaetano

Sarandöl, Emre

Sassoli, Chiara

Sato, Shuzo

Saulnier, Pierre-Jean

Savic, Lidija

Saygin, Mustafa

Sayin, Cihat Burak

Schiller, Adalbert

Schneditz, Daniel

Schnuelle, Peter

Schreiber, Nikolaus

Schwab, Sebastian

Sears, Sophie

Seck, Sidy

Seckiner, Ilker

Senturk Ciftci, Hayriye

Serafini, Gianluca

Sever, Lale

Shabaka, Amir

Shah, Ankur

Shahy, Eman M.

Shang, Jin

Sharbatdaran, Arman

Sheela, Devi C

Sheikh, Mohammad S.

Shen, Liming

Shen, Xiaogang

Shen, Yaqi

Sheng, Feng

Sheng, Wang

Sheshadri, Anoop

Shetty, Aneesha

Shima, Hisato

Shimada, Satoshi

Shinkura, Reiko

Shishido, Seiichiro

Shoop, David

Shu, Zhanjun

Shutov, Evgeny

Shvartz, Vladimir

Si, Yachen

Siddiqi, Nikhat Jamal

Siddique, Saulat

Sidoti, Anna

Sikaneta, Tabo

Silberzweig, Jeffrey

Silva Junior, Geraldo

Singh, Manisha

Singh, Manjinder

Singhai, Atin

Sinha, Rajiv

Snelson, Matthew

Sobiak, Joanna

Sofue, Tadashi

Solanki, Chetan Ram

Soliman, Karim

Soltani, Nepton

Song, Feier

Song, Guohua

Song, Jiayu

Song, Jinfang

Song, Kaixin

Song, Zhixia

Sosa-Barrios, Rosa Haridian

Spasovski, Goce

Spencer, Timothy R.

Srivastava, Swayam Prakash

Stamellou, Eleni

Staplin, Natalie

Stavroulopoulos, Aristeidis

Steele, Cortney N

Stefanidis, Constantinos

Stefanidis, Ioannis

Stepanova, Natalia

Stepien, Ewa

Strippoli, Raffaele

Stroescu, Ramona-Florina

Strohmaier, Walter Ludwig

Strohmaier, Walter Ludwig

Su, Baihai

Su, Guobin

Su, Lianjiu

Su, Ning

Subun, Chantida

Sugahara, Sho

Sumer, Ismail

Sun, Aochuan

Sun, Jing

Sun, Qihao

Sun, Wei

Sun, Zeguo

Sun, Zhaoxing

Sung, Junne-Ming

Sunilkumar, Siddharth

Suzuki, Yusuke

Swanson, Kurtis

Szabó, Éva

Szaszi, Katalin

Taheri, Saeed

Tajai, Preechaya

Takagi, Yutaka

Takahashi, Fumihiko

Takata, Tomoaki

Talakić, Emina

Tan, Hui Zhuan

Tan, Rongshao

Tan, Rui-Zhi

Tan, Ruoyun

Tanaka, Hiroshi

Tanaka, Shinji

Tang, Hailin

Tang, Jun-Ming

Tang, Ju-Yu

Tang, Shuifu

Tang, Wen

Tang, Xinfang

Tang, Yuyan

Tangpanithandee, Supawit

Taoka, Toshiaki

Tapolyai, Mihály

Teisseyre, Maxime

Tejera-Muñoz, Antonio

Telen, Marilyn J

Teo, Boon Wee

Teodorescu, Victoria

Tereshchenko, Larisa

Thammathiwat, Theerachai

Thenmozhi, P

Thevenod, Frank

Thibaudin, Damien

Thongsricome, Thana

Tian, Na

Titeca-Beauport, Dimitri

Toda, Naohiro

Tokumoto, Masanori

Tonyali, Senol

Toprak, Omer

Torisu, Kumiko

Trachtman, Howard

Trevisani, Francesco

Troyanov, Stephan

Tsai, Ming-Tsun

Tsakas, Sotirios

Tsivgoulis, Georgios

Tsujita, Makoto

Tunca, Onur

Tuncel, Altug

Tunnicliffe, David J

Tuttolomondo, Antonino

Tzanakis, Ioannis

Uchiyama, Kiyotaka

Uduagbamen, Peter Kehinde

Ueda, Hiroyuki

Uehara, Genta

Uemura, Takayuki

Ulasi, Ifeoma

Uleri, Alessandro

Unis, Amina

Upadhyay, Kiran

Urban, Matthew W.

Usuzaki, Takuma

Uwaezuoke, Samuel N

Vakhshoori, Mehrbod

Valeri, Anthony

Vazquez-Padron, Roberto I

Velarde, Victoria

Velho, Tiago R

Verhulst, Anja

Veskovic, Milena

Vijayan, Madhusudan

Vlachopanos, Georgios

Vonbrunn, Eva

Vujicic, Bozidar

Wada, Takehiko

Wada, Yukihiro

Walker, Heather

Walters, Giles

Wan, Shanshan

Wan, Xin

Wand, Ori

Wang, Baodong

Wang, Deguang

Wang, Fang

Wang, Fei

Wang, Fengying

Wang, Guo-Qin

Wang, Hai

Wang, Hongliang

Wang, Jiawu

Wang, Jincheng

Wang, Jinwei

Wang, Liang

Wang, Liansheng

Wang, Mi

Wang, Peng

Wang, Taiyao

Wang, Tingting

Wang, Weiming

Wang, Wenyun

Wang, Xiaoqin

Wang, Xiuge

Wang, Yan

Wang, Yan

Wang, Yuanyuan

Wang, Yuehua

Wang, Zhiyu

Wang, Zunsong

Wanhong, Lu

Warming, Peder Emil

Watanabe, Hirofumi

Wehrli, Felix W.

Wei, Chengguo

Wei, Qingqing

Wen, Wen

Wen, Zongmei

Weng, Zebin

Wesson, Donald E.

Widdop, Robert

Widjanarko, Nicolas Daniel

Williams, Gabrielle G

Wolfgram, Dawn F.

Wong, Chew Ming

Wong, Jiunn

Wong Meng Kit, David

Woodrow, Graham

Worawichawong, Suchin

Wu, Buyun

Wu, Chia-Chao

Wu, Chia-Chun

Wu, Chung-Kuan

Wu, Gang

Wu, Heng

Wu, Jianyong

Wu, Juan

Wu, Lingling

Wu, Ming

Wu, Shu-Yu

Wu, Xianfeng

Wu, Yong

Wu, Yuanhao

Xiao, Qin

Xiao, Rui

Xiao, Xiangcheng

Xiao-Ke Zheng, Xiao-Ke Zheng

Xiaotian, Liu

Xie, Bo

Xie, Honglang

Xie, Jingzhi

Xie, Peng

Xing, Guolan

Xing, Yan

Xiong, Lin

Xiong, Lin

Xu, Guangtao

Xu, Hong

Xu, Yan

Xu, Yili

Xu, Yunfei

Xue, Li-Jun

Xue-Rong, Wang

Yadav, Priyank

Yadav, Shikha

YalçıN, Nadir

Yalin, Serkan Feyyaz

Yamada, Shunsuke

Yamada, Shunsuke

Yamaguchi, Satoshi

Yamaguchi, Shintaro

Yamauchi, Junji

Yan, Jianyun

Yan, Jiayi

Yan, Liang-Jun

Yang, Bo

Yang, Guang

Yang, Guang

Yang, Huibin

Yang, Junlan

Yang, Min

Yang, Min

Yang, Ning

Yang, Qiongqiong

Yang, Shujun

Yang, Xiang-Hong

Yang, Xiao-Wei

Yao, Congcong

Yao, Lu

Yap, Yit-Sheung

Yapa, Harith Eranga

Yasar, Emre

Yatim, Karim

Yazawa, Masahiko

YazıCı, Halil

Ye, Nan

Ye, Wenling

Ye, Zheng

Yel, Sibel

Yessayan, Lenar

Yildirim, Fatih

Yilmaz, Akar

Yin, Jiazhen

Yin, Meng

Yin, Yan

Y J, Anupama

Yong, Kenneth

Yong, Tuck

Yongfang, Yuan

Yoshida, Ruka

Yoshida, Tadashi

Yoshimura, Yusuke

You, Li-Jun

Yu, Chen

Yu, Feng

Yu, Pei

Yu, Shengyou

Yu, Xiao-Yong

Yu, Yan

Yuan, Yanggang

Yuksel, Selcuk

Yüksel, Egrilmez Mehtap

Zaimi, Maria

Zaitone, Sawsan

Zakrocka, Izabela

Zang, Xiujuan

Zawistowski, Michał

Zeng, Guixing

Zeng, Ming

Zeng, Siyao

Zhan, Yiqiang

Zhang, Chun

Zhang, Dongliang

Zhang, Hao

Zhang, Hongliang

Zhang, Hongmei

Zhang, Jianzhong

Zhang, Jinhua

Zhang, Kang

Zhang, Lei

Zhang, Lei

Zhang, Lina

Zhang, Liuping

Zhang, Liyuan

Zhang, Pei

Zhang, Qian

Zhang, Rui

Zhang, Tie-Ning

Zhang, Wei

Zhang, Wei

Zhang, Xian K.

Zhang, Xinzhou

Zhang, Yi

Zhang, Yimin

Zhang, Zhigang

Zhang, Zhongheng

Zhao, Hanxue

Zhao, Huiping

Zhao, Jiaxi

Zhao, Jinghong

Zhao, Jun

Zhao, Long

Zhao, Peng-Yue

Zhao, Ping

Zhao, Shuan

Zhao, Wen Jing

Zhao, Xinju

Zhao, Xiujuan

Zhao, Yan

Zhao, Yuliang

Zheng, Qiyan

Zheng, Xiangyi

Zheng, Xinglong

Zheng, Zigui

Zheng, Zihe

Zhong, Zhaozhong

Zhou, Guang-Peng

Zhou, Hui

Zhou, Long

Zhou, Wenli

Zhou, Xinglei

Zhou, Yang

Zhou, Yun

Zhou, Yunping

Zhu, Jiefu

Zhu, Li

Zhu, Zewu

Zhuang, Hongjie

Zilberman-Itskovich, Shani

Zou, Hequn

Zou, Jianzhou

Zou, Xiangyu

İLeritürk, Mustafa

肖 祥

ŞEhİRlİ, Ahmet Özer

